# A Lesson in Standardization – Subtle Aspects of the Processing of Samples Can Greatly Affect Dogs' Learning

**DOI:** 10.3389/fvets.2020.00525

**Published:** 2020-08-18

**Authors:** Claire M. Guest, Rob Harris, Iqbal Anjum, Astrid R. Concha, Nicola J. Rooney

**Affiliations:** ^1^Medical Detection Dogs, Greenway Business Park, Milton Keynes, United Kingdom; ^2^US Army Research Office - Texas Tech University, Lackland, TX, United States; ^3^Milton Keynes University Hospital, Milton Keynes, United Kingdom; ^4^Animal Welfare and Behaviour Group, Bristol Veterinary School, University of Bristol, Bristol, United Kingdom

**Keywords:** dog, training, confounder, odor, standardization, processing, prostate cancer

## Abstract

Training new medical odors presents challenges in procuring sufficient target samples, and suitably matched controls. Organizations are often forced to choose between using fewer samples and risking dogs learning individuals or using differently sourced samples. Even when aiming to standardize all aspects of collection, processing, storage and presentation, this risks there being subtle differences which dogs use to discriminate, leading to artificially high performance, not replicable when novel samples are presented. We describe lessons learnt during early training of dogs to detect prostate cancer from urine. Initially, six dogs were trained to discriminate between hospital-sourced target and externally-sourced controls believed to be processed and stored the same way. Dogs performed well: mean sensitivity 93.5% (92.2–94.5) and specificity 87.9% (78.2–91.9). When training progressed to include hospital-sourced controls, dogs greatly decreased in specificity 67.3% (43.2–83.3). Alerted to a potential issue, we carried out a methodical, investigation. We presented new strategically chosen samples to the dogs and conducted a logistic regression analysis to ascertain which factor most affected specificity. We discovered the two sets of samples varied in a critical aspect, hospital-processed samples were tested by dipping the urinalysis stick into the sample, whilst for externally sourced samples a small amount of urine was poured onto the stick. Dogs had learnt to distinguish target aided by the odor of this stick. This highlights the importance of considering every aspect of sample processing even when using urine, often believed to be less susceptible to contamination than media like breath.

## Introduction

When starting to train dogs to detect new emerging target odors, organizations are often faced with a challenge of procuring adequate numbers of both target samples, and suitably matched controls (1, 2). As a consequence, they are often forced to choose between using small numbers of training samples and risking the dog's learning to identify individual samples (rather than the target vs. control distinction) or sourcing samples from multiple places. The latter choice, whilst aimed at increasing the possibility of dogs' learning to discriminate the target odor, risks the possibility that, if samples come from limited sources, and are not processed identically, dogs may learn to distinguish target from control, based on a confounding factor. To mitigate this, organizations training dogs, aim to standardize all aspects of the collection, processing and sample presentation, however even with the greatest care, there may remain subtle factors which differ and which dogs can potentially learn to use to discriminate during training. This can lead to artificially inflated performance rates during training, which are not replicable when novel samples from another source are presented for training or blind testing.

There is a real challenge when training dogs to learn complex odors in complex environments, especially when those training them are not aware what the odor signature is, as is the case for many medical detection tasks [e.g., (1)]. What's more, there is variation in both targets and controls, and the aim of training is to ensure that the animal's identification responses are being controlled by disease-related Volatile Organic Compounds (VOCs) rather than other volatiles unique to the individual who provided the sample (2). It is important to ensure that training conditions are conducive to this “concept formation.”

Studies of explosives have shown that increasing the variation in training samples of TNT (3) and gunpowder (4) improved generalization by the dogs and hence increased the likelihood of “concept formation.” Odor profiles associated with disease are complex and are presented within numerous background odors. Gas chromatography-mass spectrometry studies of urine collected from prostate patients, identified over 500 potentially relevant VOCs amongst a total of over 9,000 (5). As Edwards et al. (2) advise, ideally a wide variety of positive samples with a single commonality: positive disease status and likewise, a wide variety of negative samples with a single commonality – negative disease status – is needed. However, Edwards et al. (2) also point out, one of the largest challenges in olfactory detection of human disease is sample availability.

Since dogs can identify individual human odors [e.g., (6)] and retain individuals in their memory (1), if one starts to train on a small number of samples, they run the risk of dogs simply learning to recognize whether each person's samples are rewarded or not. Hence, training organizations may be forced to seek larger numbers of samples and controls that may not be sourced identically for example using more than one hospital or other sources. It is commonly acknowledged that sourcing human target and control samples from non-matched sources presents the risk that dogs learn to discriminate based on a confounder or cross contamination (7). Whilst such issues are widely acknowledged when using breath as a medium (7), the risk with more stable media such as urine is less widely known, and therefore believed to be less of a risk.

This was the situation when Medical Detection Dogs (MDD) the UK's leading medical detection dog charity, first started to train dogs to detect prostate cancer from urine. Proof of principle studies had suggested that dogs can be trained to detect prostate cancer from urine (8, 9). But when MDD started to train for this, they were faced with the challenge of having small initial numbers of samples, especially controls, supplied by a single source. Below we describe the training that was carried out, the issue that emerged and our logical and systematic effort to identify and overcome this.

## Methods

The study received Ethical Approval from North West - Lancaster Research Ethics Committee (Ref:15/NW/0527).

### Sample Collection

Samples were collected from Milton Keynes University Hospital (MKUH), both positive samples and age- and symptom-matched controls from men attending urological outpatients' clinics and to supplement control sample numbers, men and women from external Medical Detection Dog (MDD) events. All participants were over 18 years, had no previous history of malignancy (urological or non-urological), were not undergoing dialysis, nor had a diagnosis of HIV or Hepatitis (except Hepatitis A).

All urine samples were believed to be collected and processed in the same way. Participants were provided with a collection pot and plastic gloves and asked to urinate directly into the pot. Samples were then handled by the experimenter or nurse who tested for urine composition (presence of UTI, diabetes, and kidney disorders) using a urinalysis stick (Siemens Multistix 10SG), labeled the sample and placed it in a portable freezer, before being frozen in the hospital's freezer or, in the case of external samples, MDDs freezer. Samples taken at the hospital, were stored for up to 6 months, and then following the patient's diagnosis by biopsy, cystology or MRI, were classified as positive for prostate cancer or negative controls. Since these control patients likely had other urological conditions, they were classified as “unhealthy” and were only used in the most advanced stage of discrimination training (Stage 4) when they were age-matched and symptom-matched to cancerous targets.

### Sample Processing

A Standard Operating Procedure was followed to avoid cross contamination. Each consenting participant's whole sample was defrosted and spun in a vortex machine for 10 s; separated into several 1 m samples, each decanted into a 1.75 glass vial and marked with an anonymised code. All aliquots of the same code were stored in the same zip-lock bag in the -20C freezer. Samples were selected and defrosted on the day of training and then placed in a refrigerator for no longer that 1 h, before being decanted into 60 ml polystyrene pots for training. Each aliquot was used once during only one training session.

### Dogs

The six dogs were all female, there were two Labrador Retrievers, two Labrador crosses, one Cocker Spaniel and one Wire-haired Hungarian Vizsla. At the start of training, dogs ranged in age from 14 to 54 months old. Their training involved four stages which involved gradually increasing the number of samples presented and the subtlety of the difference between target and control. So, dogs started with a small number of controls that were all healthy, the diversity of controls was gradually increased and ultimately included “unhealthy” controls, that may have had a urological condition other than prostate cancer (as described above). Whilst the positive samples were all from men, the controls included females. Although females never occurred in the targets, in the early stages of training we included wide ranging control samples varying in multiple aspects, to encourage the dog to learn the important discriminatory cue (9).

### Dog Training – Stage 1

All training was performed in a dedicated room in the Bio-Detection building at Medical Detection Dogs, UK. Dogs were initially taught to recognize the target scent using search games. The target scent was paired with a food or play reward. Gradually the dogs were trained to follow a more formalized search pattern, when-upon samples were presented in either a four-stand line-up or an eight-position carousel into which stainless steel plates each containing a polystyrene pot were placed.

Training used a 100% reward protocol. Dogs were encouraged to search all vials and when a target sample was encountered to show a trained alert behavior (sit and stare), but to show no response to control samples. When a dog showed a correct positive response, it was rewarded with an audible clicker (as a secondary reinforcer) followed by food or a play reward, whilst a dog showing an incorrect alert was ignored, and encouraged to keep searching. Dogs were also trained to carry out blank runs in which no target samples were presented, whereupon they were rewarded for searching all the apparatus but not showing any alerting behaviors.

Due to a paucity of control samples initially dogs were trained over a 70-day period (individual dogs ranged from 53 to 70 days), using 21 positive samples [100% male; aged 28–80 years; all confirmed prostate cancer positive of Gleason score 3+3 to 4 + 3; (10)] collected from Milton Keynes Hospital Urological clinic and 215 control samples collected from external events, by self-declared healthy volunteers (65% male aged 50–80 years). Over this period, dogs on average received 312 (± 75.5) presentations of positive samples (ranging from 230 to 419) and on average 1,088 (± 182.6: range 768–1,260) of controls. Each presentation was a separate aliquot decanted from a sample, and each aliquot was used during only one training session, although during this session, multiple dogs were usually presented with the same sample numerous times. New controls and targets were gradually introduced throughout this training phase.

Dogs performance in all training sessions was recorded using a computer data base, and sessions were filmed using CCTV for later analysis, if required. Whenever presented with a sample, the dog's response was classified as correct (trained alert to a target and no response to a control) or incorrect. When dogs exhibited a hesitation when encountering a sample, but no full alert, since the dogs were in the training phase, this was treated as an alert so in response to a target was classified as a true positive and rewarded whilst when in response to a control it was classified as a false positive and the behavior was ignored.

Over the initial Stage 1, all six dogs were seen to be performing well, with sensitivities (% of positive samples correctly identified) ranging from 92.2 to 94.5% (averaging 93.5%) and specificities (% of the control samples that were ignored) ranging from 78.2 to 91.9% (averaging 87.9%; Table 1).

**Table 1:** Demographics and training performance of each of the six female dogs.

**Dog name**	**Breed**	**Stage 1: Initial training**		**Stage 2: Training including new healthy MKUH control samples**		**Stage 4: Post investigation re-training using MKUH healthy and unhealthy controls**	
		**% Sensitivity**	**% Specificity**	**% Sensitivity**	**% Specificity**	**% Sensitivity**	**% Specificity**
Florin	Labrador retriever	94.2	87.5	93.4	67.8	92.5	81.4
Karry	Labrador cross	94.5	89.9	92.4	83.3	86.5	77.9
Kim	Labrador cross	92.2	91.9	87.1	77.1	83.3	71.9
Kiwi	Labrador retriever	93.9	89.3	75.3	65.5	57.1	88.9
Martha	Cocker spaniel	92.7	78.2	91.9	43.1	Dog Rejected	
Midas	Wire-haired hungarian vizsla	93.7	90.5	90.7	67.1	85.7	79.7

*Shaded cells highlight those dogs rejected from the programme due to training issue mid- or post study*.

### Dog Training – Stage 2

The dogs then progressed to Stage 2, when-upon 79 new control samples and 13 target samples were added to the training pool. The controls were samples that had been collected from volunteers (staff, relatives and friends), 48 male and 23 female and 8 unknown, ranging in age from 18 to 79 years, attending the same clinic as the initial targets and internal hospital recruitment events. All volunteers were self-declared healthy. These new samples were presented in combination of the external MDD controls over a 10-week training period, averaging 482 (± 188.9: range: 221–707) control and 148 (± 123.9 range 122–192) target sample presentations per dog. When training progressed to include these hospital-sourced controls, a noticeable decrease in performance, was seen particularly in specificity which now averaged only 67.3% (43.1–83.3%; Table 2).

**Table 2:** Seven urine samples procured for the systematic investigation of confounding factors.

**Sample**	**Vesicle storage site**	**Sample collection site**	**Processing methods**	**Sex**	**Age in years**
1	MDD	MKUH	MKUH	Female	38
2	MKUH	MKUH	MKUH	Female	38
3	MKUH	MKUH	MDD	Male	25
4	MKUH	External	MKUH	Male	19
5	MDD	MKUH	MKUH	Female	34
6	MKUH	MKUH	MKUH	Female	34
7	MDD	MKUH	MDD	Female	30

Examination of the training data showed that the drop in performance was specific to the new control samples, in response to which the dog showed a large number of false positive responses, leading to a reduction in measured specificity. Novel samples of both controls and targets had been gradually added throughout training so it was unlikely a response to novel samples [e.g., (11)]. Since the new control samples came from healthy volunteers of a similar age range to the initial external samples, we had no reason to assume that they were any harder to discriminate than those used in Stage 1. This suggested that in Stage 1 dogs had learnt to distinguish the original targets from control samples, on the basis of a factor other than disease state.

Now, in order to rectify the dog's training and ensure optimal sample collection and processing and hence training and performance in the future, we aimed to identify which confounding factor the dogs had used. Alerted to a potential training issue, we carried out a methodical and sequential investigation into all factors which could potentially vary between the hospital and external samples. We used a small number of carefully chosen samples to complete this investigation, avoiding wasting precious training samples.

The training team suspected that the processing or storage at the two sites may have differed. We therefore embarked upon an investigative phase. We observed the processes at both sites from collection to delivery to the dog and discussed the procedures with the hospital nursing team to obtain any clues as to systematic differences between sites. We were assured that there were no systematic differences in: type of gloves used to handle the sampling vesicles; disinfectant used to clean areas, or length of time for which samples were stored in the cool box, prior to being placed in the freezer between the two sites.

However, we identified three potential differences:

Vesicle storage site - location where storage pot was long-term stored (MDD or MKUH);

Sample collection site– place where sample was collected (MDD external events or MKUH);

Processing method: at the two sites (MDD or MKUH)

We next meticulously and systematically investigated which of these factors was the causal issue using new samples and all six training dogs.

### Stage 3 - Investigative Stage

We recruited five control human volunteers to provide urine: three females and two males. Four gave urine samples at the hospital, one at an external venue. Two participants gave two samples each, one in MKUH and one MDD stored collection vesicles. In total, we procured seven samples (Table 2) presenting different combinations of the suspected confounding factors.

We presented these samples to each of the dogs (within an assortment of other targets and the control samples) a number of times (between 11 and 91 presentations per sample) in order to identify which factor was most linked to high rates of false positive alerts. By recording the number of incorrect alerts to each sample (false positives) performed by each dog, we could carry out statistical analysis to identify which factor was most responsible and hence the major confounder. The effects on specificity were estimated from logistic regression models including these three factors and allowing for differences in performance between dogs. The effects were expressed as odds ratios, and least squares means were estimated for each factor.

## Results

The results showed that individual dogs vary widely in their specificity (Table 3; *p* < 0.0001). Storage site had a marginally significant effect, with samples in MKUH storage vesicles resulting in significantly lower specificity than MDD samples, but that the effect of processing method had the biggest impact (Chi squared = 14.4 *p* = 0.0001). Control samples which underwent Medical Detection Dog's (MDD) processing, were more likely to be correctly ignored than samples undergoing Milton Keynes University Hospital's (MKUH) processing (OR = 4.32), as were those placed in vesicles stored at MDD (OR = 2.11), whilst externally sourced samples were slightly less likely to be ignored (OR = 0.55). The response to each of these factors varied between individual dogs (Table 4).

**Table 3:** Results of Logistic regression exploring effect of number of factors on specificity of dog's response to control samples.

**Effect**	**DF**	**Odds ratio**	**Wald chi-square**	***P***
Dog ID	5		26.92	<0.0001
Vesicle storage site (base category MKUH)	1	2.11 (1.03, 4.33)	4.17	0.0411
Sample collection site (base category MKUH)	1	0.55 (0.25, 1.2)	2.25	0.1332
Processing method (base category MKUH)	1	4.32 (2.03, 9.21)	14.39	0.0001

**Table 4:** Percentage specificity of dog's response when each factor is compared (hesitations without a full alert were classified as alerts and hence constituted a true positive on a target sample and a false positive on a control).

**Dog's name**	**Vesicle storage site - location where storage pot was long-term stored**		**Sample collection site– place where sample was collected**		**Processing method**	
	**MDD**	**MKUH**	**External**	**MK**	**MDD**	**MKUH**
Florin	88.9	45.0	14.3	80.0	94.7	53.6
Karry	85.7	83.3	66.7	89.3	100	75.6
Kim	95.2	76.2	76.9	87.3	90.0	83.3
Kiwi	52.4	72.7	40.0	63.0	66.7	40.0
Martha	66.7	15.4	15.4	66.7	83.3	27.3
Midas	92.1	59.4	50.0	83.9	96.2	65.9

Once the processing sites was implicated as the most important factor, the team watched the sample handling post-patient, the cleaning of equipment and observed that they varied only in a subtle aspect of their processing; whilst hospital-sourced samples were tested using a urinalysis stick dipped into the sample, externally-sourced samples were tested by pouring a small amount the sample onto the stick. Therefore, only the hospital-sourced training samples contacted the urinalysis stick and hence, the dogs had likely learnt to distinguish target from non-target aided by the odor of this stick.

### Dog Training: Stage 4

Based on this knowledge, we modified our subsequent training (Stage 4) and processing to ensure standardization (e.g., all samples were decanted and applied to a urinalysis stick externally). Also, having identified the main confounder, the four remaining dogs (two were rejected prior to this stage due to ongoing training issues) were now trained intensely, with a large number of sample presentations per day and concentrating only on teaching the distinction of malignant vs. non- malignant whilst ignoring the previously learnt processing factor. Matched controls from individuals sampled at the same MKUH clinic, but subsequently diagnosed as having non-malignant organ-specific conditions and no history of cancer, were also included at this stage. With very large numbers of presentations of both controls (664–1,016 per dog) and targets (196–376 per dog), including 143 familiar and 217 novel samples, we progressively trained three of the dogs to ignore the processing method and alert based only on disease state, the fourth dog failed to respond to this training and was therefore also rejected.

It is noteworthy, that whilst this rehabilitation training served to teach three of the six dogs to categorize samples based on disease state and they achieved high levels of performance (71.9–88.9% specificity: Table 1), three dogs failed either during Stage 3 (Kiwi and Martha), or during Stage 4 (Kim) to be retrained and required to be rejected from future trials.

## Discussion

This study supports previous findings [e.g., (8, 9)], that dogs can be trained to detect prostate cancer, but shows that even within a population of all female, similarly selected and trained dogs, individuals showed very different levels of both sensitivity and specificity. Once dogs were seemingly well-trained on initial samples, we saw a decrease in specificity when new control samples were added. This demonstrates that even when trained on target and control samples that were apparently identically collected, processed and stored, dogs had learnt to discriminate targets from non-target, not by the intended disease state, but by a confounding factor.

By using carefully chosen samples with each combination of potential confounders and employing statistical analysis, we were able to identify the most likely causal factor. The results of our logistic regression of training data indicated that the biggest effect on performance at the discrimination task was dog ID, highlighting individual differences between the dogs, each varying widely in their specificity. Collection vesicle storage site had a marginally significant effect, but the sample processing method had by far the greatest impact. This showed that control samples which underwent Medical Detection Dog's (MDD) processing, were more likely to be correctly ignored than samples undergoing Milton Keynes University Hospital's processing, as were those placed in vesicles stored at MDD, whilst externally sourced samples were slightly less likely to be ignored.

The analysis highlighted that a confounder associated with the processing was likely inflating the dogs' overall specificity during the initial training. The dogs appeared to have learnt to use a cue to discriminate samples from one another, and this cue was not only disease state, but something associated with the site at which the processing occurred. The actual reason was not obvious as the Standard Operating Procedure was believed to be identical in all cases. It was only by watching and discussing with the onsite healthcare team, that the most critical elements of the process were identified. We discovered the two sets of samples varied in a subtle aspect of their processing; hospital-sourced samples were tested by dipping the urinalysis stick into the sample, for MDD processed samples, a small amount of urine was poured onto the stick. We conclude that dogs had likely learnt to distinguish target aided by the odor of this stick.

It can be argued, that if samples need to be sourced from more than one location, ideally clinical processes should be replicated and an external person should watch the processing and minimize the possibility of confounders pre-training. Potential factors should be identified and eliminated from the outset. However, this can be an onerous task, especially in medical settings when samples which would normally be collected from consented patients by health care professionals who may rotate daily. However, this case study highlights the importance of considering and monitoring every aspect of sample collection, processing and delivery when using a limited number of collection locations, even when using urine for dog training. Although it may have seemed trivial to clinical health care professionals (involved in patient consent and sample collection) whether a dipstick was placed into the fresh urine sample after patient production, or a drop is taken from the urine, we have shown that for dogs working on a highly complex discrimination task, this aspect had a significant effect. Studies of dog training show that given multiple possible cues by which dogs can solve a training task, dogs will learn to use the cue(s) most salient and accessible to them [e.g., (12)], and here it appears that the altered odor created by the dipstick was that cue. Urine was previously believed to be less susceptible to cross contamination and processing effects than more volatile media such as breath (13). It is widely acknowledged that ambient VOC's can contaminate breathe samples [e.g., (7)], but here we demonstrate that even for a liquid medium there is significant risk of cross contamination so standardized processing of samples is essential. It is not known exactly what effect the dipstick inclusion had on the urine sample or how it changed the odor, but the canine performance indicated that it was a significant factor in learning discrimination. Interestingly the extent of the effect varied between dogs.

There is currently limited research examining factors that affect a dog's propensity to generalize or discriminate odors. The balance between generalization and discrimination in odor recognition is affected by target odor molecular structure (14), as structurally similar molecules compete and activate overlapping receptors, making these compounds harder to discriminate (15) and the olfactory threshold may vary for different compounds (16). The tendency to discriminate may also vary with the individual dog's olfactory acuity (8), and with training and reward protocols [e.g., (12, 17)]; and here we suggest also individual personality differences in the dog. This is an area important for future study.

Our results show a moderate significant effect of vesicle storage site, suggesting that ambient atmosphere may have contaminated the storage pots and had some effect on the dogs, but since sampling site did not exert a significant effect, we have no evidence that changes such as time pre- freezing, freezer temperature or differences in procedures when samples are moved from the clinic to the freezer (which varied between sites) were used by dogs to categorize the samples. However, this may be because in our case, the processing methods, and odor of the urinalysis stick was the most salient cue, and we cannot rule out that if the processing cue were absent (due to standardization), the dogs would not have learnt to discriminate based on sample collection site or vesicle storage location to a greater extent. Given the potential for subtle aspects to affect training, we suggest future studies should aim to standardize all aspects and that papers reporting dog detection results should state clearly where and how all samples have been collected and how audits are carried out to ensure that internal and external sites achieve identical processing. Historically this has not always been the case [see (6)].

As pointed out by Edwards et al. (2) the validity of performance and results are threatened when systematic differences between positive and control samples (other than disease status) are present during training phases. Here there was a systematic difference which was pinpointed by a systematic investigation. When training a complex signature in a complex background, we need to ensure dogs learn to accurately discriminate disease state only. This is best achieved by using completely matched samples from a single site. Controls should be from the same clinical environment as the targets, and ideally collected at the same time since ambient VOCs may vary from time to time even within the same environment. If such standardization is impossible and confounders are unavoidable, then we need to maximize the variation in them e.g., by using multiple collection methods, locations and processing methods for both targets and controls to ensure the dogs learn to categorize based only on the target factor: disease state (7). But, when training for novel diseases presented only in a limited number of patients or with a paucity of initial control sample as seen here, it is often not possible and, in such cases, we have demonstrated how when training anomalies arise, a thorough investigative stage is extremely valuable.

Here due to a lack of initial controls we needed to source control samples from additional sites. This was important, in order to avoid the issue identified by Elliker et al. (1) when training two dogs to detect prostate cancer, dogs appeared to memorize the samples and hence not generalize to new samples when presented in double-blind testing. Canine memory is an important consideration when training with a limited number of samples and again points to the necessity for larger training sets collected from the same source or if not, multiple varied sources (1, 18).

The study also demonstrated the value of continually monitoring performance throughout training, in order to be able to rapidly identify if a training problem develops or the performance of dogs is being affected by a confounding factor. Ideally this should be accompanied by rigorous blinding throughout training, so that human cues do not present an additional confounder (7). The electronic monitoring system at MDD allowed us to continuously monitor performance and to analyse individual accuracy on a rolling basis. All training sessions were recorded using an internally developed database system MDD-Olfactory Performance Recording Application (OPRA). Each session was filmed using CCTV and stored on a protected drive for later analysis. Use of this footage, and the analytical methods described here, allowed us to identify that there was a problem, and a systematic and methodical investigative phase allowed the route of the problem to be pinpointed and subsequent remedial training carried out. The technology also aided objective performance measurement and decision-making regarding individual dogs. Trainers often become heavily invested in the dogs with which they work and are challenged to make objective decisions about performance and accuracy of individuals. Being able to review and collaboratively discuss footage, can also allow consensus decisions e.g., before withdrawing a dog from training.

It is noteworthy that of the six dogs starting this trial, three were rejected as a result of the them learning to distinguish based on a confounding factor and trainers being unable to re-train the correct categorization within a reasonable time frame. Two dogs were rejected after Stage 3, and one during and one after Stage 4. Although showing great initial aptitude for the task, having learnt the incorrect discrimination cue, in spite of large numbers of presentations and positive reward-based training, these dogs failed to learn the correct association, and systematic search errors and behavioral issues ensued. In such cases, trainers often find it easier to start with a new dog than to rectify the problem, which highlights the potentially great costs of issues in initial odor training. Interestingly, the training data shows that the extent to which the confounder was used to discriminate samples varied with dog. Whilst Table 3 suggests that Kim predominantly learnt the disease vs. control distinction, as intended, Martha for example relied heavily on the difference in processing in her decision making. Further research into these individual differences is required.

The importance of minimizing potential confounding cues which the dogs can use in place of the intended categorization feature, is obvious in this medical context. However, this concept applies equally well when training dogs for narcotics and explosives and other targets when-upon dogs often learn, for example, that training and hence rewards only ever occur outside an operational scenario or when seniors trainers as well as handlers are present (19). However, our study shows that when subtle differences apparently lead to training issues, systematic analytic methods can be employed to identify and subsequently rectify the problem.

## Data Availability Statement

Data are available at the University of Bristol data repository, data.bris, at https://doi.org/10.5523/bris.3mw2y33y7a1j32v3r7r77t953z.

## Ethics Statement

Retrospective routine dog training data collected during Medical Detection Dogs usual training operations was analyzed. Ethical review and approval for animal use was not required according to institutional guidelines. Owner consent was not required as the dogs were the property of Medical Detection Dogs at the time.

## Author Contributions

CG, RH, and IA: concept formation. AC, CG, and RH: conducted investigative work. RH and NR: analysis. NR, CG, and RH: manuscript preparation. All authors: edits and approval.

## Conflict of Interest

CG, RH, AC, and NR were all employed by Medical Detection Dogs the charity which trained and own the dogs. However, since the study described training issues and their resolution. We do not see this as a conflict of interest. The remaining author declares that the research was conducted in the absence of any commercial or financial relationships that could be construed as a potential conflict of interest.

## References

[1] Elliker KR Sommerville BA Broom DM Neal DE Armstrong S Williams HC. Key considerations for the experimental training and evaluation of cancer odour detection dogs: lessons learnt from double-blind, controlled trail of prostate cancer detection. BMC Urol. (2014) 14:22. 10.1186/1471-2490-14-22 24575737 PMC3945616

[2] Edwards TL Browne CM Schoon A Cox C Poling A Animal olfactory detection of human diseases: guidelines and systematic review. J Vet Behav. (2017) 20:59–73. 10.1016/j.jveb.2017.05.002

[3] Goldblatt A Gazit I Grinstein D Terkel J The Olfactory System and Olfaction: Implications for REST. (2011). Available online at: https://www.gichd.org/fileadmin/GICHD-resources/rec-documents/REST-Nov2011.pdf (accessed 03 August 2020)

[4] Oxley JC Waggoner LP Detection of explosives by dogs. In: Marshall M Oxley J, editors. Aspects of Explosives Detection. Amsterdam: Elsevier (2009). p. 27–40.

[5] Gao Q Su X Annabi MH Schreiter BR Prince T Ackerman A. Application of urinary volatile organic compounds (VOCs) for the diagnosis of prostate cancer. Clin Genitouri Cancer. (2019) 17:183–90. 10.1016/j.clgc.2019.02.003 30853355

[6] Schoon GAA De Bruin JC. The ability of dogs to recognize and cross-match human odours. (1994) *Forensic Sci Int*. 69:111–118 10.1016/0379-0738(94)90247-X 7813994

[7] Jezierski T Walczak M Ligor T Rudnicka J Buszewski B. Study of the art: canine olfaction used for cancer detection on the basis of breath odour. Perspectives limitation. J Breath Res. (2015) 9:027001. 10.1088/1752-7155/9/2/027001 25944810

[8] Cornu J-N Cancel-Tassin G Ondet V Girardet C Cussenot O. Olfactory detection of prostate cancer by dogs sniffing urine: a step forward in early diagnosis. Eur Assoc Urol. (2010) 59:197–201. 10.1016/j.eururo.2010.10.006 20970246

[9] Taverna G Tidu L Grizzi F Torri V Mandressi A Sardella P. Olfactory system of highly trained dogs detects prostate cancer in urine samples. J Urol. (2015) 193:1382–7. 10.1016/j.juro.2014.09.099 25264338

[10] Gleason DF. Classification of prostatic carcinomas. Cancer Chemother Rep. (1966) 50:125–8. 5948714

[11] Pirrone F Albertini M Olfactory detection of cancer by trained sniffer dogs: a systematic review of the literature. J Vet Behav. (2017) 19:105–17. 10.1016/j.jveb.2017.03.004

[12] DeChant MT Ford C Hall NJ. Effect of handler knowledge of the detection task on canine search behavior and performance. Front Vet Sci. (2020) 7:250. 10.3389/fvets.2020.00250 32528982 PMC7266931

[13] Amundsen T Sundstrøm S Buvik T Gederaas OA Haaverstad R. Can dogs smell lung cancer? First study using exhaled breath and urine screening in unselected patients with suspected lung cancer. Acta Oncol. (2014) 53:307–15. 10.3109/0284186X.2013.819996 23957595

[14] DeGreeff LE Simon AG Peranich H Holness HK Frank K Furton KG. Generalization and discrimination of molecularly similar odorants in detection canines and the influence of training. Behav Proces. (2020) 177:104148. 10.1016/j.beproc.2020.104148 32464153

[15] Mershin A Wassie A Maguire Y Kong D Zhang S Moran P Methods and Apparatus for Artificial Olfaction. U.S Patent: US9140677B2 (2015).

[16] Walker DB Walker JC Cavnar PJ Taylor JL Pickel DH Hall SB Naturalistic quantification of canine olfactory sensitivity. Appl Anim Behav Sci. (2006) 97:241–54. 10.1016/j.applanim.2005.07.009

[17] Walczak M Jezierski T Górecka-Bruzda A Sobczynska M Ensminger J Impact of individual training parameters and manner of taking breath odor samples on the reliability of canines as cancer screeners. J Vet Behav. (2012) 7:283–94. 10.1016/j.jveb.2012.01.001

[18] Williams M Johnston JM Training and maintaining the performance of dogs (*Canis familiaris)* on an increasing number of odor discriminations in a controlled setting. Appl Anim Behav Sci. (2002) 78:55–65. 10.1016/S0168-1591(02)00081-3

[19] Gazit I Goldblatt A Terkell J. The role of context specificity in learning: the effects of training context on explosives detection in dogs. Anim Cogn. (2005) 8:143–50. 10.1007/s10071-004-0236-9 15449101

